# Novel *MEF2C* point mutations in Chinese patients with Rett (−like) syndrome or non-syndromic intellectual disability: insights into genotype-phenotype correlation

**DOI:** 10.1186/s12881-018-0699-1

**Published:** 2018-10-30

**Authors:** Jiaping Wang, Qingping Zhang, Yan Chen, Shujie Yu, Xiru Wu, Xinhua Bao, Yongxin Wen

**Affiliations:** 1 Department of Pediatrics, Peking University First Hospital, No 1, Xi’anmen Street, Xicheng District, Beijing, 100034 China; 2 Department of neurology, Harbin children’s hospital, Harbin, 150010 Heilongjiang Province China

**Keywords:** *MEF2C*, Rett (−like) syndrome, Non-syndromic intellectual disability, Genotype-phenotype correlation

## Abstract

**Background:**

*MEF2C* (Myocyte-specific enhancer factor 2C) has been associated with neurodevelopmental disorders. This study aimed at delineating the clinical profiles of *MEF2C* gene mutations.

**Methods:**

In total, 112 Chinese patients with intellectual disability (ID) were recruited, including 44 patients presented with Rett syndrome (RTT) or RTT-like syndrome, and 68 patients with non-syndromic ID. Targeted next-generation sequencing (NGS) was performed. Detailed clinical information was collected.

**Results:**

Five heterozygous *MEF2C* gene mutations were identified, of which three were novel. The *MEF2C* mutant rate was 4.5% (5/112) in total, and 6.8% (3/44) in the RTT (−like) cohort. All patients with *MEF2C* gene mutation presented with cognitive impairment, gross motor delay, speech disorder and autistic features. Four patients had epilepsy, which responded well to antiepileptic drugs. One female was diagnosed with classical RTT, two females with RTT-like syndrome, and two males with non-syndromic ID. Generally, the phenotype of two males with relatively downstream mutations (c.565C > T, p.Arg 189*; c.766C > T, p.Arg 256*) was milder than that of three females with upstream mutations (c.48C > G, p.Asn16Lys; c.334G > T, p.Glu112* and c.403-1G > T).

**Conclusions:**

Our findings expanded the current understanding of the consequences of *MEF2C* dysfunctions, especially *MEF2C* point mutations. *MEF2C* mutations are associated with a broad clinical spectrum, ranged from classical RTT to non-syndromic ID. Through our study, it can be inferred that there is correlation between the phenotype and *MEF2C*-genotype, the mutation site. Overall, the *MEF2C* gene mutational analysis should be performed in ID cohort, especially in patients with features overlapped with RTT.

**Electronic supplementary material:**

The online version of this article (10.1186/s12881-018-0699-1) contains supplementary material, which is available to authorized users.

## Background

*MEF2C* (Myocyte-specific enhancer factor 2C), a member of MEF2 subfamily, is a transcriptional activator binding specifically to the MEF2 element, which has pivotal role in myogenesis, hematopoiesis, neurogenesis and synaptogenesis [1, 2]. Especially, *MEF2C* is crucial for normal neuronal development, distribution, and electrical activity in the neocortex [3]. *MEF2C* haploinsufficiency caused by either large deletions encompassing *MEF2C*, or intragenic mutations of *MEF2C*, is associated with neurodevelopmental disorders [4].

In this study, we performed targeted next-generation sequencing (NGS) in Chinese patients with intellectual disability (ID) of unknown causes. *MEF2C* mutations were identified in five patients, including three females with RTT or RTT-like features, and two males with non-syndromic ID. The phenotypes and genotype of *MEF2C* mutation in Chinese ID patients were reported here.

## Methods

### Patients

In total, 112 Chinese patients with ID were enrolled into this study from August 2015 to March 2018, including 68 females and 44 males, aged from 13 months to 12.5 years. Among them, 25 girls were initially diagnosed with RTT (21 with typical RTT, 2 with Han-RTT, 1 with Zappella variant of RTT and 1 with congenital RTT), 16 females and 3 males with RTT-like syndrome. Mutations in *MECP2, CDKL5* and *FOXG1* have been ruled out in the patients of RTT (−like) cohort. The other 68 patients presented with non-syndromic ID. Metabolic or mitochondrial disorders, central nervous system infection or other known etiologies were excluded.

Detailed clinical information including clinical manifestation, electroencephalogram (EEG), magnetic resonance imaging (MRI), and family history, etc., was collected. Genomic DNA was extracted from peripheral leukocytes of the patients and their parents. This study was approved by the Medical Ethics Committee, Peking University First Hospital. Written informed consent was obtained from the parents of the patients.

### Targeted next-generation sequencing

A genetic panel which is related to ID was designed, containing 512 genes (supplemental material, Additional file 1: Table S1) in total. Especially, genes associated with RTT (−like) syndrome were included. Library preparation including end repair, adapter ligation and PCR enrichment were carried out as recommended by the NEBNext Rfast DNA Fragmentation and Library Prep Set for Ion Torrent. Targeted regions were captured with a customized SeqCap panel (Roche). The enriched libraries were sequenced on an Ion Torrent Proton sequencer with 200 bp single end read. Signal processing and base calling were carried out using Torrent Suite 5.04 software. Reads were aligned to the human reference genome build UCSC hg19 using Tmap. Variants were called using the variant caller from Torrent Suite.

Variants were annotated using ANNOVAR (http://annovar.openbioinformatics.org/en/latest/), assessment of the variants were carried out following the ACMG guidence. Common sites with population allele frequency above 5% according to dbSNP 138, 1000 Genome Project, esp6500si and ExAC databases were excluded. Rare loss-of-function variants were selected for follow-up analyses. Selected variants with depth lower that 20X and allelic fraction lower than 30% were manually reviewed with IGV. Functional variants annotated by HGMD, Clinvar or predicted to be deleterious by SIFT and Polyphen2 were regraded as candidates.

## Results

### Molecular analysis

Causative gene mutations were identified in 14 patients of our RTT (−like) cohort, with detection rate of 31.8% (14/44), incluing 3 *MEF2C* mutations. In the non-specific ID group, 9 patients (13.2%, 9/68) were identified with pathogenic mutations, including 2 *MEF2C* mutations. The wild type and mutant allele of *MEF2C* is approximately at 1:1 (ranging from 39.1 to 47.2%), so mosaicism were not considered.

**Fig. 1:**
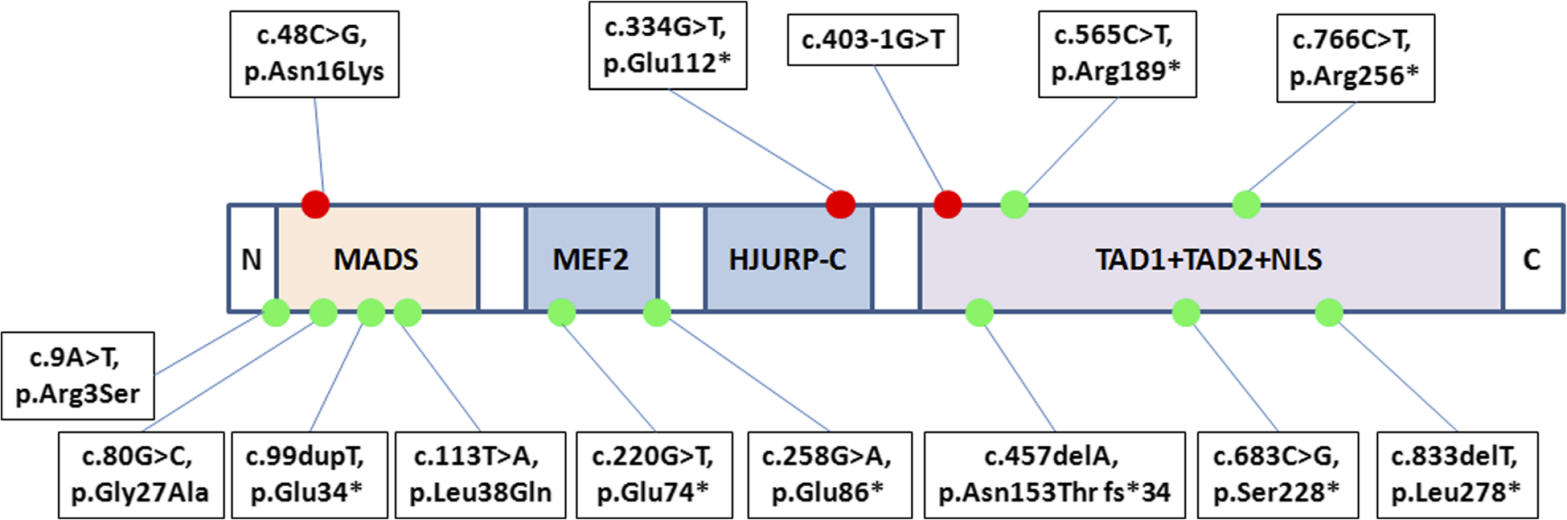
The structure of the *MEF2C* protein and the location of *MEF2C* mutations. Red dots: Novel mutations found in this study. Green dots: Reported mutations. The upper line: Five *MEF2C* mutations identified in this study. The lower line: Nine *MEF2C* mutations that has been described previously. N, N-terminus; MADS, MCM1, agamous, deficiens, serum response factor; MEF2, myocyte enhancer factor 2; TAD, transcriptional activation domain; NLS, nuclear location signal; C, C-terminus

Five heterozygous mutations in *MEF2C* were identified in three females with RTT (−like) syndrome and two males with non-syndromic ID. The *MEF2C* mutant rate was 4.5% (5/112) in total, whereas 6.8% (3/44) and 2.9% (2/68) in Rett (−like) cohort and non-syndromic ID group, respectively. These five mutations included three nonsense mutations, one splicing site mutation, and one missense mutation. Three *MEF2C* mutations (c.48C > G, p.Asn16Lys; c.403-1G > T; c.766C > T, p.Arg256*) arose de novo, and the other two (c.565C > T, p.Arg189*; c.334G > T, p.Glu112*)were of unknown origin, as the parental DNA was not available. Among these mutations, two mutations, c.565C > T, p.Arg189* and c.766C > T, p.Arg256*, were reported pathogenic, the other three mutations, c.48C > G, p.Asn16Lys; c.334G > T, p.Glu112*; and c.403-1G > T, were novel. They were predicted as pathogenic by Mutationtaster, the missense mutations, c.48C > G, p.Asn16Lys, was predicted as pathogenic by PolyPhen-2 and SIFT as well. A schematic representation of the *MEF2C* protein, including the mutations identified in this study as well as the mutations previously reported, is shown in Fig. 1.

### Clinical profiles of patients with *MEF2C* mutations

Patient 1 is a girl aged 5 years and 9 months, the first child of a healthy nonconsanguineous couple. Her psychomotor milestones were profoundly delayed, with raising head at 8 months, sitting alone at 1 year old, and still unable to walk by herself at 5 years and 9 months. Poor eye contact, hand clapping, hand wringing and bruxism were observed at 1 year old, followed by deterioration of hand skills. Epileptic attack occurred at 20 months old, and she responded to valproate (VPA), oxcarbazepine (OXC), and topiramate (TPM) combined therapy. Seizure free was achieved at 5.5 years old. EEG demonstrated spike-slow waves at right medial and posterior temporal, with generalization. MRI (1 year old) revealed enlargement of frontal subarachnoid space. Above manifestations led to the diagnosis of typical RTT. However, mutational analysis of *MECP2, CDKL5* and *FOXG1* was negative. Through this study, a de novo missense *MEF2C* mutation, c.48C > G, p.Asn16Lys, was identified, which was a novel mutation.

Patient 2 is a 2.5 years old girl presented with RTT-like ID. she displayed profound psychomotor retardation, with controlling head at 5 months, sitting alone at 8 months. She remained unable to walk independently and still had no speech at 2.5 years old. She developed stereotypic hand movements and bruxism at 2 years of age. No seizure was reported, but there was epileptiform discharge on EEG at age of 2 years. Brain MRI revealed high T_1_ and T_2_ signal at posterior horn of bilateral ventricle. A nonsense mutation, c.565C > T, p.Arg189*, of *MEF2C* was discovered, a known disease causing mutation (https://www.ncbi.nlm.nih.gov/clinvar/).

Patient 3 is a 23-month-old girl who presented with RTT-like features. Feeding difficulties caused concern at 3 months of age. No epilepsy was observed, though she had medical history of febrile convulsions at 9 months old. There was significant delay in her developmental milestones, without obvious retrogression. She could sit alone at 1 year old, and walk at 23 months of age, with abnormal gaits. Unmeaningful language began at 12 months. She also presented with poor eye contact, stereotypic actions, breathing disturbance, and sleeping abnormalities. She suffered from recurrent respiratory infections frequently after 15 months of age. MRI revealed hypomyelination at 1 year and 10 months of age. A novel nonsense mutation, c.334G > T, p.Glu112*, in *MEF2C* was identified.

Patient 4 was a boy aged 7 years and 8 months. He achieved the gross motor developmental milestones somewhat delay, with rising head at 1 year old, sitting alone at 1 year and 2 months, and walking at 1.5 years. Lack of speech was another problem, that he still cannot speak a single word so far (7 years and 8 months). He was always immersed in his own world, showed little interest to the others, and lacked eye-contact. Febrile seizures attacked at 1 year, which turned into afebrile seizures at 2 years old. Partial seizures occurred 1~ 2 times per month, lasted few minutes to more than half an hour. The epilepsy was fever-sensitive. VPA was used at 2.5 years of age, and no seizures occurred after 4 years of age. EEG at 2.5 years displayed (multi-) spike and slow waves at right occipital region, with slow rhythm on the background. MRI at 3 years of age was normal. A novel mutation, c.403-1G > T, of *MEF2C* was identified, which arose de novo.

Patient 5 was a boy of 6 years and 4 months. He presented with developmental delay, with rising head at 7 months, sitting alone at 1 year, and walking at 2 years old. Language delay was a main problem, that he could only speak one to two words, such as “give me”, “I want”. He also displayed some autistic behaviors, including occasionally repetitive hand movements, no eye-contact and no interest to others. Febrile seizures initially occurred at 8 months of age, then attacked 2–3 times per year. Seizure-free was achieved for 4 months with levetiracetam (LEV) and VPA. MRI at 4 years old revealed long T_1_ and T_2_ signal around bilateral ventricle, and a septum pellucidum cyst. EEG was normal at 4 years of age. A known pathogenic de novo mutation, c.766C > T, p.Arg256*, in *MEF2C* was discovered.

The genetic data and clinical information from the five patients with *MEF2C* mutations were summarized in Table 1.

**Table 1:** Clinical information of patients with *MEF2C* (NM_001193350) mutation

ID	Patient 1	Patient 2	Patient 3	Patient 4	Patient 5
Sex	F	F	F	M	M
Mutation	c.48C > G, p.Asn16Lys	c.565C > T, p.Arg189*	c.334G > T, p.Glu112*	c.403-1G > T	c.766C > T, p.Arg256*
Origin	de novo	mother WT; father unknown	father WT; mother unknown	de novo	de novo
Age	5y 9mo	2 y, 6 mo	2y 4mo	7y 8mo	6y 4 mo
Sz (age of onset)	Y (1y 10mo)	N	Y (9mo)	Y (1y)	Y (8mo)
Sz types	PS	–	FS	FS, PS, SE	FS
Response to AEDs	Seizure free for 4 months, with OXC, VPA, TPM	–	–	seizures free after 4 y, with VPA	seizures free 6y, with LEV, VPA
Motor development	Head control (8 mo), sit (12 mo), cannot walk	Head control (5 mo), sit (8 mo), cannot walk, head circumstance 48.5 cm (2y)	Head control (2 mo), sit (12 mo), walk (23 mo), abnormal gaits, head circumstance 45.3 cm (2y 4mo)	Head control (1y), sit (1y 2 mo), walk (1 y, 6 mo)	Head control (7 mo), sit (1y), walk (2y), head circumstance 52 cm (6y 4mo)
Cognitive outcome	Severe ID	Severe ID	Severe ID	Severe ID	Severe ID
Language	N	N	Unmeaningful murmur	N	1 to 2 words
Poor eye-contact	Y	Y	Y	Y	Y
Repetitive behaviour	Y	Y	Y	Occassionlly	Occassionally
Poor hand skills	Y	Y	Y	Normal	Normal
Hypotonia	Y	Y	Y	N	N
Family history	N	N	N	N	N
EEG	Sharp-slow waves at right medial and posterior temporal, followed by generalization (2y, 11mo)	NA	Normal (2y, 4mo)	Sharp-and-slow waves around Pz(4y,1mo)	Normal (4 y)
MRI	enlargement of frontal subarachnoid space (1 y)	Long T_1_ and T_2_ signal at posterior horn of bilateral ventricle.	Hypomyelination (1y, 10 mo)	Normal (3y)	Long T_1_ and T_2_ signal around bilateral ventricle, septum pellucidum cyst (4y)
Other symptoms	hypalgesia	_	Feeding difficulty, sleeping disturbance, irritability, recurrent respiratory infection, bruxism	No interest to others	No interest to others

F, female; M, male; Y, yes; N, no; NA, not avaliable; ID, intellectual disability; VPA, valproate; LEV, levetriacetam; OXC, oxcarbazepine; TPM, topiramate; y, year; mo, month; AEDs, antiepileptic drugs

## Discussion

In this study, five *MEF2C* mutations were identified in RTT (−like) syndrome or non-syndromic intellectual disability patients, of which three mutations were novel. The *MEF2C* gene was originally identified as the phenocritical gene in the 5q14.3 microdeletion syndrome [5, 6]. Consecutively, intragenic deletions/insertions and point mutations of *MEF2C* were discovered in patients with neurodevelopmental disorders, including intellectual disability and epilepsy [7]. As most patients were detected having multi-gene microdeletions involved *MEF2C*, the phenotype of *MEF2C* gene mutation is not well recognized [8, 9]. Up to now, only 14 point mutations in *MEF2C* have been described in patients with neurological disorders [7, 10–13], including mutations discovered in this study, as is shown in Fig. 1. Our findings provide support for delineating the clinical features of patients with *MEF2C* point mutations, as well as the genotype-phenotype relationship.

*MEF2C* gene, located at 5q14, encodes myocyte enhancer-binding factor 2C, a transcription factor that is indispensable in early neuroprogenitor development [2, 14]. The human *MEF2C* protein consists five core structural domains, including MADS (MCM1, agamous, deficiens, serum response factor), myocyte enhancer factor 2 (MEF2), transcriptional activation domain 1 (TAD1), transcriptional activation domain 2 (TAD2), and nuclear localization signal (NLS) [15]. The MADS domain contains 56 amino acids, the MEF2 domain starts from amino acid 57 to 86, and the HJURP-C domain consists of 30 amino acids, while the stereostructure of rest domians remains unknown. The main role of MADS domain and the adjacent *MEF2C* domain is to mediate dimerization and DNA binding, whereas the rest domains act as the transcriptional activator [15]. The *MEF2C* mutations are scattered through the whole *MEF2C* protein, and there’s no hot mutant region (Fig. 1).

Typical clinical manifestations in patients with *MEF2C* gene mutations encompass global developmental delay, intellectual disability, absent speech, poor eye-contact and various minor brain anomalies on MRI [11]. A subset of patients with *MEF2C* haploinsufficiency phenotypically resembles RTT, including psychomotor stagnation, stereotypical behavior, poor hand skills, etc., [4, 7, 16] Our findings basically coincided with previous description. All patients of our cohort presented with cognitive impairment, speech disorder, gross motor delay and lack of eye-contact. MRI was abnormal in four out of five patients, with diverse anomalies, including enlargement of frontal subarachnoid space, hypomyelination, abnormal signals at posterior horn of bilateral ventricle or bilateral ventricle. Among them, one female manifestated as classical RTT and other two females displayed RTT–like phenotype, whereas two males presented with non-syndromic ID.

The overwhelming majority of patients who had macro-deletions partially or entirely encompassing the *MEF2C* gene, cannot walk independently. However, including 3 of our cases (patients 3–5), 7 (7/14, 50%) patients with *MEF2C* point mutations acquired the walking abilities [12, 17]. It indicates that *MEF2C* point mutations might cause milder phenotypes than large intragenic deletions or completely deletions. Additionally, the phenotype of patient 4 (c.565C > T, p.Arg 189*) and patient 5 (c.766C > T, p.Arg 256*) was milder than that of patient 1 (c.48C > G, p.Asn16Lys), patient 2 (c.334G > T, p.Glu112*) and patient 3 (c.403-1G > T). As it was shown in Fig. 1, that the later three mutations located at the upstream, whereas the other two mutations located at the downstream of the *MEF2C* protein, which suggested that the phenotypic severity may be associated with the mutation site.

Stereotypic behaviors, such as head rocking, hand-mouthing, hand clapping and wringing were reported in some patients with *MEF2C* mutations [7]. In our cohort, repetitive hand movements were obvious in the three females, whereas it was just occassionally observed in two male cases. Meanwhile, the three females only had limited hand skills, while it was basically normal in both male patients. Besides, bruxism, breathing and sleeping disturbance were also presented in the three females of RTT (−like) cohort. The reason accounted for the different clinical feature in male and female patients with *MEF2C* gene mutation need to be further studied.

More than half patients with *MEF2C* haploinsufficiency experienced epilepsy [18]. Seizures generally arised during infancy or early childhood, which were commonly triggered by fever. Tonic-clonic seizures, myoclonic seizures were the most common seizure types, partial seizures, infantile spasms, atypical absence and febrile seizures were also described. Most epilepsy patients responded well to anti-epileptic drugs, whereas refractory in a few cases were also reported [18]. In this study, four (4/5, 80%) patients developed epilepsy within one year old. Febrile seizures were occurred in all of them, partial seizures were reported in two patients, and one patient had history of epileptic status. Seizure-free was achieved in all of them by mono- or multi-antiepileptic drugs (AEDs) therapy.

Mild to severe hypotonia was reported in most of the patients [12]. In our cohort, severe hypotonia was observed in three females. MRI showed nonspecific abnormalities in four of our patients, including hypomyelination, enlarged ventricles, abnormal signal on posterior horn of bilateral ventricle, just as that was reported in the literature [5].

Recently, *MEF2C* mutations were founded in patients with heart disorders, including patent ductus arteriousus (PDA), double outlet right ventricle (DORV), ventricular septal defect (VSD) and dilated cardiomyopathy (DCM), accompanied with or without neurological symptoms [19–21]. All patients of our cohort had no medical history of heart diseases. As DCM is usually adult-onset, a longer term of ultrasonic cardiogram monitoring is necessary for patients with *MEF2C* mutations. There was no relationship between the genotype and phenotype with or without heart disorders and/or neurological symptoms. The clinical heterogeneity might indicated the partly incomplete penetrance.

## Conclusions

Our findings expanded the spectrum of point mutations of *MEF2C* gene, and also showed some relationship between phenotype and *MEF2C*-genotype. The different clinical features in male and female patients with *MEF2C* gene mutation need to be verified in a larger sample. *MEF2C* gene mutational analysis was recommended in patients with ID, especially in the RTT (−like) cohort without mutations in traditional RTT genes (*MECP2, CDKL5, FOXG1*) .

## Additional file

Additional file 1: **Table S1.** List of 512 candidate genes. (DOCX 20 kb)

## Abbreviations

AEDs: antiepileptic drugs
DCM: dilated cardiomyopathy
DORV: double outlet right ventricle
EEG: electroencephalogram
ID: intellectual disability
LEV: levetiracetam
NGS: next-generation sequencing
OXC: oxcarbazepine
PDA: patent ductus arteriousus
TPM: topiramate
VPA: valproate
VSD: ventricular septal defect
