# Supplementary material for: Novel MEF2C point mutations in Chinese patients with Rett (−like) syndrome or non-syndromic intellectual disability: insights into genotype-phenotype correlation
Source: BMC Med Genet. 2018 Oct 30;19:191. doi: 10.1186/s12881-018-0699-1 (PMC6208086; doi:10.1186/s12881-018-0699-1)
Supplement: Supplementary file 1 — Table S1. List of 512 candidate genes. (DOCX 20 kb) [file 12881_2018_699_MOESM1_ESM.docx]

**Table S1 List of 512 candidate genes**

| AARS | ABCB7 | ABCC8 | ABCD4 | ACADSB | ACTB | ACY1 | ADGRV1 |
| --- | --- | --- | --- | --- | --- | --- | --- |
| ADK | ADRA2B | ADSL | AFG3L2 | AGAT | AHI1 | AKT1 | ALDH5A1 |
| ALDH7A1 | ALG1 | ALG11 | ALG13 | ALG3 | AMACR | AMER1 | AMPD2 |
| AMT | ANKLE2 | AP3B2 | APTX | ARG1 | ARHGAP31 | ARHGEF9 | ARL13B |
| ARSA | ARSE | ARV1 | ARX | ASAH1 | ASPM | ATCAY | ATIC |
| ATM | ATN1 | ATP13A2 | ATP1A2 | ATP1A3 | ATP2A2 | ATP2B3 | ATP6AP2 |
| ATRX | ATXN1 | ATXN10 | ATXN2 | ATXN3 | ATXN7 | ATXN8 | B9D1 |
| BDNF | BEAN | BEAN1 | BOLA3 | BRAT1 | BRP44L | BTD | C19ORF12 |
| C5ORF42 | CACNA1A | CACNA1D | CACNA1H | CACNB4 | CACNG2 | CAD | CASC5 |
| CASR | CC2D2A | CDK5 | CDK5RAP2 | CDK6 | CDKL5 | CENPE | CENPJ |
| CEP104 | CEP135 | CEP152 | CEP290 | CEP41 | CERS1 | CHD2 | CHMP1A |
| CHRNA2 | CHRNA4 | CHRNB2 | CLCN2 | CLN3 | CLN5 | CLN6 | CLN8 |
| CLP1 | CNNM2 | CNTN2 | CNTNAP2 | COA5 | COASY | COG7 | COH1 |
| COQ8 | COQ8A | COX14 | COX6B1 | CPA6 | CPS1 | CPT2 | CRH |
| CSPP1 | CSTB | CTSA | CTSD | CTSF | CYB5R3 | D2HGDH | DBH |
| DCX | DENND5A | DEPDC5 | DHFR | DIP2B | DMPK | DNAJC5 | DNM1 |
| DNM1L | DOCK6 | DOCK7 | DPYD | DRD3 | DRD5 | DTNBP1 | EBP |
| ECM1 | EEF1A2 | EFHC1 | EHMT1 | EIF2B1 | EIF2B2 | EIF2B3 | EIF2B4 |
| EIF2B5 | EIF4G1 | ELP4 | EMX2 | EPB41L1 | EPM2A | EVC | EXOSC3 |
| EXOSC8 | FADD | FAM123B | FASTKD2 | FGF12 | FGF14 | FKTN | FLNA |
| FMR1 | FOLR1 | FOXG1 | FOXP1 | FOXP2 | FOXRED1 | FRRS1L | FTL |
| FXN | GABBR2 | GABRA1 | GABRA6 | GABRB1 | GABRB2 | GABRB3 | GABRD |
| GABRG2 | GAL | GAMT | GATM | GBA | GCK | GCSH | GFAP |
| GJC2 | GLB1 | GLDC | GLRA1 | GLUL | GNAO1 | GOSR2 | GPHN |
| GRIN1 | GRIN2A | GRIN2B | GRIN2D | GRIN3B | GRN | GSS | GUF1 |
| GYS1 | HAX1 | HCN1 | HCN2 | HFE | HNF1B | HNRNPU | HP |
| HRAS | HSD17B10 | HSD17B4 | HTT | HUWE1 | IDH2 | IDS | IER3IP1 |
| IFNG | IKBKG | INPP5E | INS | IQSEC2 | ISG15 | ITPA | ITPR1 |
| JMJD1C | JPH3 | KATNB1 | KCNA1 | KCNA2 | KCNB1 | KCNC1 | KCNC3 |
| KCNH1 | KCNJ10 | KCNJ11 | KCNMA1 | KCNQ1 | KCNQ2 | KCNQ3 | KCNT1 |
| KCTD7 | KDM5C | KIAA0556 | KIAA0586 | KIF11 | KIF1A | KIF1BP | KIF7 |
| KNL1 | KRAS | KRIT1 | L2HGDH | LAMB1 | LAMC3 | LBR | LGI1 |
| LIAS | LMBRD1 | LMNB2 | LMX1B | MAN1B1 | MANBA | MAPK10 | MBD5 |
| MCCC2 | MCPH1 | MDH2 | MECP2 | MED17 | MEF2C | MFSD2A | MFSD8 |
| MKS1 | MLC1 | MMAA | MMAB | MMACHC | MMADHC | MOCS1 | MOCS2 |
| MPC1 | MRE11 | MRE11A | MTHFR | MTOR | MTR | MTRR | NDE1 |
| NDN | NDUFA1 | NDUFA11 | NDUFA12L | NDUFAF1 | NDUFAF2 | NDUFAF3 | NDUFAF4 |
| NDUFAF5 | NDUFB3 | NDUFS1 | NDUFS2 | NDUFS4 | NDUFS6 | NDUFV1 | NDUFV2 |
| NECAP1 | NEU1 | NF1 | NF2 | NHLRC1 | NHS | NIPBL | NOP56 |
| NOTCH3 | NPHP1 | NPR2L | NPRL2 | NPRL3 | NRCAM | NRXN1 | NTNG1 |
| NUBPL | OFD1 | OPHN1 | PAFAH1B1 | PAH | PAK3 | PANK2 | PANR2 |
| PCDH19 | PCLO | PDE6D | PDGFB | PDGFRB | PDHA1 | PDXP | PDYN |
| PEX1 | PEX2 | PEX26 | PGAP2 | PGAP3 | PGK1 | PHC1 | PHF6 |
| PHGDH | PIGA | PIGL | PIGN | PIGO | PIGT | PIGV | PIGW |
| PIGY | PLA2G6 | PLCB1 | PLEKHG4 | PLP1 | PLPBP | PNKP | PNPO |
| POLG | POMGNT1 | PPOX | PPP2R2B | PPT1 | PRDM8 | PRICKLE1 | PRICKLE2 |
| PRKCG | PRNP | PROC | PRODH | PROSC | PRRT2 | PTCHD1 | PTF1A |
| PTPN4 | PUS1 | QDPR | RAB39B | RAD21 | RANBP2 | RARS2 | RELN |
| RERE | RHOBTB2 | ROGDI | RPGRIP1L | RPIA | RTTN | RYR3 | SASS6 |
| SATB2 | SCARB2 | SCN1A | SCN1B | SCN2A | SCN3A | SCN8A | SCN9A |
| SDHA | SEPSECS | SERPINI1 | SETBP1 | SETX | SGCE | SHANK3 | SHH |
| SHROOM4 | SIAT9 | SIK1 | SIL1 | SIX3 | SLC12A5 | SLC13A5 | SLC16A1 |
| SLC17A5 | SLC19A3 | SLC1A2 | SLC1A3 | SLC20A2 | SLC25A12 | SLC25A15 | SLC25A22 |
| SLC26A3 | SLC26A4 | SLC2A1 | SLC35A2 | SLC46A1 | SLC6A1 | SLC9A6 | SLC9A9 |
| SMARCA1 | SMC1A | SMC3 | SNAP29 | SNIP1 | SNRPN | SOBP | SPATA5 |
| SPTAN1 | SPTBN2 | SPTLC2 | SRPX2 | ST3GAL3 | ST3GAL5 | STIL | STK11 |
| STRADA | STS | STX1B | STXBP1 | SUOX | SYN1 | SYNGAP1 | SYP |
| SZT2 | TBC1D24 | TBCE | TBP | TCF4 | TCN2 | TCTN1 | TCTN2 |
| TCTN3 | TDP1 | TGIF1 | TGM6 | TMEM138 | TMEM165 | TMEM216 | TMEM231 |
| TMEM237 | TMEM67 | TOE1 | TPP1 | TREM2 | TREX1 | TSC1 | TSC2 |
| TSEN2 | TSEN34 | TSEN54 | TTBK2 | TTC21B | TTC38 | TTPA | TUBA1A |
| TUBB2B | TUBGCP6 | TWNK | TYROBP | UBA5 | UBE3A | UROD | VPS13B |
| VPS53 | VRK1 | WDR45 | WDR62 | WWOX | XK | XPR1 | ZBTB18 |
| ZDHHC15 | ZEB2 | ZFX | ZIC2 | ZNF238 | ZNF335 | ZNF41 | ZNF423 |
